# Maternal TSH-receptor antibodies predict fetal/neonatal hyperthyroidism in pregnancies with a history of Graves disease

**DOI:** 10.1210/clinem/dgag118

**Published:** 2026-03-18

**Authors:** Langeza Saleh, Marco W J Schreurs, Eric Boersma, Hemmo A Drexhage, Sabine M P F de Muinck Keizer-Schrama, Willy Visser

**Affiliations:** Department of Obstetrics and Gynaecology, Division of Obstetrics and Prenatal Medicine, Erasmus Medical Center Rotterdam, 3015 CN Rotterdam, the Netherlands; Department of Immunology, Laboratory Medical Immunology, Erasmus Medical Center Rotterdam, 3015 CN Rotterdam, the Netherlands; Department of Cardiology, Thorax Center, Erasmus Medical Center Rotterdam, 3015 CN Rotterdam, the Netherlands; Department of Immunology, Erasmus Medical Center Rotterdam, 3015 CN Rotterdam, the Netherlands; Department of Endocrinology, Sophia Children's Hospital, Erasmus Medical Center, 3015 CN Rotterdam, the Netherlands; Department of Internal Medicine, Division of Pharmacology and Vascular Medicine, Erasmus Medical Center Rotterdam, 3015 CN Rotterdam, the Netherlands

**Keywords:** pregnancy, TSH receptor antibody titers, fetal hyperthyroidism, neonatal hyperthyroidism

## Abstract

**Context:**

Transplacental transfer of thyrotropin receptor antibodies (TRAbs) during pregnancy may cause fetal/neonatal thyroid dysfunction. Current guidelines recommend a universal TRAb titer threshold, irrespective of gestational age or assay platform. However, evidence supporting trimester-specific and assay-specific thresholds is lacking.

**Objective:**

This work aimed to determine whether trimester-specific TRAb levels predict fetal/neonatal thyroid dysfunction and to establish assay-specific cutoff values for clinical risk stratification.

**Methods:**

In this prospective, multicenter, observational cohort study (2010-2023), maternal TRAb titers were measured using the TRAK human assay on the BRAHMS Kryptor Compact Plus analyzer. Settings were tertiary and secondary care centers (n = 44) across the Netherlands. Patients included women with a history of (n = 500) or active (n = 178) Graves disease. Main outcome measures included biochemically confirmed fetal/neonatal hyperthyroidism or hypothyroidism.

**Results:**

Trimester-specific TRAb titer thresholds predicted risk with high accuracy. In women with a history of Graves disease (n = 500), trimester-specific cutoffs of 25-, 19-, and 6-fold the upper limit of normal (ULN) showed a negative predictive value of 100%. In women with active Graves (n = 178), cutoffs of 10-, 12-, and 10-fold the ULN showed sensitivities of 89%, 86%, and 83%. Neonatal hyperthyroidism occurred despite low or declining maternal TRAb levels in mothers treated with antithyroid drugs.

**Conclusion:**

Trimester-specific TRAb thresholds were higher than the universal ULN multipliers commonly applied in clinical practice, and differed by gestational age and disease status. Application of a single ULN-based threshold titer across different TRAb assays may result in misclassification (ie, overestimation of fetal and neonatal Graves disease) and consequently in unnecessary and resource-intensive fetal surveillance. This indicates that TRAb thresholds are assay specific and require platform-specific clinical validation, even when assays are calibrated against a common international standard.

In Graves disease the body produces thyrotropin receptor antibodies (TRAbs), and this may lead to hyperthyroidism during pregnancy (1). Transplacental transfer of TRAbs may induce fetal or neonatal thyroid dysfunction, particularly hyperthyroidism (and more rarely hypothyroidism in the presence of blocking antibodies). Elevated maternal TRAb titers substantially increase the risk of fetal or neonatal thyroid dysfunction, highlighting the need for accurate screening and risk stratification (2, 3). While neonatal hyperthyroidism occurs in approximately 1% to 5% of pregnancies with active Graves disease, the risk in women with a history of Graves disease, especially those who became euthyroid after definitive therapy, remains less well defined but is generally considered rare (4).

In the Netherlands, trimester-based TRAb testing is routine; evidence supporting its predictive value remains limited, particularly in early pregnancy and across different TRAb assays (5, 6). Evidence from smaller studies and one meta-analysis has linked elevated third-trimester TRAb titers with an increased risk of fetal/neonatal hyperthyroidism (4, 7, 8). However, these studies are limited by small sample sizes and the absence of trimester-specific TRAb titer thresholds, which are essential to predict unaffected pregnancies and those at risk. Additionally, the use of different TRAb assays in studies may contribute to inconsistent results.

In clinical practice, fetal surveillance is commonly guided by a universal TRAb threshold expressed as a multiple of the assay-specific upper limit of normal (ULN). This threshold was largely derived from studies using first- and second-generation TRAb assays, rather than third-generation assays. A recent study employing a third-generation automated TRAb assay (Roche) reported that maternal third-trimester TRAb concentrations of approximately 4.6 to 4.7 times the ULN were required to achieve 100% sensitivity for the prediction of neonatal thyrotoxicosis (9). This supports the view that each TRAb assay requires separate assay validation to demonstrate suitability for its intended purpose. The common practice of measuring TRAbs for the first time in the third trimester could contribute to missing early-onset fetal hyperthyroidism. Finally, research of TRAb titer thresholds to date has largely focused on women with active Graves.

We conducted a large-scale study using a single standardized assay system to evaluate the predictive value of trimester-specific TRAb titer thresholds in identifying fetal/neonatal hyperthyroidism or hypothyroidism in patients with a history or active Graves. Additionally, we established trimester-specific TRAb titer thresholds to enable clinicians to more effectively rule in or rule out fetal/neonatal complications. These findings aim to refine current screening practices and enhance the precision of risk stratification, paving the way for improved outcomes in pregnancies complicated by Graves disease.

## Materials and methods

### Study design

This prospective, observational, investigator-initiated study was conducted at 44 Dutch centers, enrolling pregnant women with either active or a history of Graves. All participants provided written informed consent, and the study protocol received approval from the local research ethics committee (MEC 211.008/2002/33) at each participating hospital.

### Patients and participants

Inclusion criteria comprised women aged 18 years or older, who had a history of Graves or were diagnosed with active Graves. Patients with a history of Graves were defined as women previously treated with antithyroid drugs, radioiodine I-131, or surgery who had achieved euthyroid status with or without thyroxine (T4) treatment at the time of enrollment. Active Graves referred to women with Graves hyperthyroidism or those receiving antithyroid drugs during pregnancy because of active Graves disease. Exclusion criteria encompassed multiple pregnancies or missing fetal/neonatal diagnosis.

### Definitions

Fetal and neonatal thyroid dysfunction was classified on the basis of biochemical thyroid function testing. The primary study end point was overt fetal or neonatal hyperthyroidism, defined by a suppressed thyrotropin (TSH) concentration in combination with an elevated free T4 (fT4) level, in the presence of elevated maternal TRAbs. Clinical and ultrasound findings, including fetal tachycardia or goiter, were assessed during follow-up, but were not sufficient to define the study end point in the absence of abnormal thyroid function tests. In this manuscript, the term *fetal/neonatal hyperthyroidism* is exclusively used when referring to TRAb-mediated fetal or neonatal hyperthyroidism, irrespective of treatment, or TRAb-mediated hypothyroidism due to blocking antibodies. To produce trimester-specific TRAb threshold and receiver operating characteristic curve (ROC) analyses, only cases of overt biochemical hyperthyroidism were included; cases with suppressed TSH and normal fT4 were classified separately and were not used in the analysis of the primary study end point.

For neonatal diagnosis, TSH and fT4 were interpreted according to gestational age and day after birth, based on the reference values established at Erasmus MC, Rotterdam, the Netherlands. Subclinical hyperthyroidism was characterized by low TSH with normal fT4 levels, while overt hyperthyroidism was indicated by low TSH and high fT4 levels. Subclinical hypothyroidism was defined by elevated TSH levels with normal fT4 levels, whereas overt hypothyroidism was marked by high TSH and low fT4 levels. Neonates who achieved euthyroid status (normal TSH and fT4 levels) by the tenth day post partum were classified as transient. Persistent conditions were defined when thyroid function did not normalize by the tenth day postpartum. Late-onset conditions were defined as postpartum manifestations that developed later during follow-up.

### Routine care

All women included in the study underwent routine care that consisted of blood sampling at their respective hospitals and received treatment from their own physicians according to local/national protocol. Fetal heart rate was carefully monitored, and all fetuses underwent a comprehensive ultrasound examination to evaluate the thyroid gland. Following delivery, cord blood samples were collected; all neonates were examined by pediatricians to assess for the clinical presence of neonatal hypothyroidism or hyperthyroidism, and blood samples were collected on day 2, day 4, and days 7 to 10 after delivery for determination of fT4 and TSH and treatment was provided if necessary.

### Study blood sampling and measurement of thyrotropin receptor antibodies, free thyroxine, and thyrotropin levels

Maternal serum samples were collected during the 3 trimesters of pregnancy. Additionally, umbilical cord blood samples were obtained at birth, and neonatal blood samples were collected on day 2, day 4, and between days 7 and 10 post partum. All samples were stored at −80 °C after collection. TRAb titers (in maternal serum samples), fT4, and TSH levels (in umbilical cord samples) were determined batchwise at the end of the study using chemiluminescence assays in the central laboratory of the Erasmus MC, Rotterdam, the Netherlands. Quantification of (total) serum TRAb titers for all participants was carried out using the TRAK human assay on the BRAHMS Kryptor Compact Plus analyzer (Thermo Fisher Scientific), according to the manufacturer's instructions. This assay uses the human monoclonal TRAb M22 (RRID:AB_2892140). In a subset of 28 individuals, a comparative analysis was conducted between the TRAK human assay and the enzyme-linked immunosorbent assay anti-TSH-R assay on the Phadia250 analyzer (Thermo Fisher Scientific), which is based on the same monoclonal antibody. The normal TRAb threshold titer for the Kryptor assay is less than 1.1 IU/L, while for the Phadia assay, the normal TRAb threshold titer is less than 2.9 IU/L. fT4 and TSH were measured by using Vitros ECi (Ortho Clinical Diagnostics).

### Statistical analysis

Continuous variables appeared to have a nonnormal distribution (visual inspection and Shapiro-Wilk test) and are therefore presented as median (25th-75th percentile, interquartile range [IQR]), whereas differences between history and active Graves were compared using the Mann-Whitney *U* test. Categorical data are presented as numbers (percentages) and were analyzed using the chi-square test, or Fisher exact test when expected cell counts were less than 5.

Logistic regression was applied to examine the association between TRAb titers (as a continuous variable) and neonatal outcomes for women with a history and active Graves separately. We report odds ratios (ORs) with 95% CI. Subsequently, for both cohorts, we visually present the relation between trimester-specific TRAb threshold titers and the sensitivity and 1–specificity for fetal/neonatal hyperthyroidism (ROC curve). We determined the TRAb threshold titers that maximize the probability to rule out cases of fetal/neonatal hyperthyroidism (thus, that maximize sensitivity).

To evaluate the comparability of TRAb titers between the BRAHMS Kryptor and Phadia TBII assay systems, we first constructed a Bland-Altman plot to assess clinical agreement, including bias and 95% limits of agreement. This method allows for evaluation of whether two assays can be used interchangeably in practice. In addition, linear regression was performed to assess the strength of correlation between the two assays.

To enable practical application of the Kryptor-derived TRAb threshold titer in settings using the Phadia platform, we calculated a conversion factor by dividing Kryptor values by their corresponding Phadia measurements. This yielded a mean factor of 1.5 (SD 0.84), establishing the relationship Kryptor = 1.5 × Phadia.

Not all women had TRAb measurements available in all 3 trimesters. This was primarily due to missing blood samples or because some participants were enrolled in the study only during the second or third trimester. As a result, TRAb titers were available in 463, 431, and 563 women in the first, second, and third trimester, respectively. Missing values are indicated in the relevant tables.

*P* less than .05 was considered statistically significant. Data analysis was performed using IBM SPSS Statistics (IBM Corporation, version 25) and GraphPad Prism version 9.

## Results

### Baseline characteristics

Of the 678 included pregnancies, 500 (74%) involved women with a history of Graves, whereas 178 (26%) had clinically active Graves (Tables 1 and 2).

**Table 1:** Maternal characteristics according to maternal diagnosis of a history of or active Graves

	History of Graves		Active Graves	
**Number of patients**	**500**	Missing	**178**	Missing
**Age** (IQR), y	34 (31-36)	20	32 (29-35)*^a^*	7
**Primigravida** (n, %)	159 (32.0)	3	45 (25.3)	1
**Nullipara** (n, %)	205 (41.4)	5	69 (38.8)	1
**Treatment,** 1st/2nd/3rd trimester (n)		7/13/19		2/6/11
No treatment	126/117/115		21/39/64	
Thyroxine	367/367/361		—/—/1	
Thyroxine and antithyroid drugs	0/3/5		21/7/6	
Antithyroid drugs	—/—/—		134/126/96	

Bold values indicate total number of participants (N) per group.

Abbreviation: IQR, interquartile range.

^a^Comparison between active and history of Graves with *P* less than .05.

**Table 2:** Fetal/neonatal outcomes according to maternal diagnosis of a history of or active Graves

	History of Graves		Active Graves	
**Number of patients**	**500**	Missing	**178**	Missing
**Girls** (n, %)	240 (49.1)	11	81 (48.5)	11
**GA at delivery** (wk + d in median, IQR)	39 + 4 (38 + 2-40 + 4)	3	39 + 5 (38 + 3-40 + 5)	1
**Delivery 24-37 wk** (n, %)	35 (7.0)*^a^*	2	21 (11.9)	1
**Birth weight** (g in median, IQR)	3360 (3018-3750)	7	3328 (2996-3704)	8
**Weight centile <10** (n, %)	69 (14.0)	7	20 (11.8)	8
**Apgar score <7 < 5 min** (n, %)	8 (1.6)	10	4 (2.3)	7
**Pregnancy outcome** (n, %)				
Ectopic pregnancy	—		1 (0.6)	
First trimester pregnancy loss	6 (1.2)		2 (1.1)	
Second trimester pregnancy loss	1 (0.2)		4 (2.2)	
Intrauterine fetal demise	1 (0.2)		2 (1.1)	
Neonatal death	1 (0.2)		1 (0.6)	

Bold values indicate total number of participants (N) per group.

Abbreviation: IQR, interquartile range.

^*a*^ *P* less than .05.

In the women with a history of Graves, 74% used levothyroxine in the first trimester. A small subset required combined therapy with antithyroid drugs to manage suspected fetal hyperthyroidism: 3 women in the second trimester and 5 in the third.

Among women with active Graves, 88% used antithyroid drugs in the first trimester, declining to 61% in the third trimester. Notably, a proportion of women in this group received nonstandard combination therapy with both levothyroxine and antithyroid drugs: 21 (12%) in the first trimester, 7 (4%) in the second, and 6 (4%) in the third.

Gestational age at delivery was similar between women with a history of and active Graves (39 weeks and 4 days vs 39 weeks and 5 days, respectively). Preterm delivery was, however, more frequent in patients with active Graves (11.9% vs 7.0%; *P* = 0.05), as was the incidence of fetal/neonatal hyperthyroidism (6.7% vs 2.6%; *P* = 0.01).

### Thyrotropin receptor antibody titers in maternal serum samples throughout pregnancy

Across all trimesters, TRAb titers were consistently and markedly lower in women with a history of Graves compared to those with active disease (*P* < 0.001) (Table 3). In the first trimester, the median TRAb titers were 1.1 IU/L (IQR 0.3-3.0) and 4.0 IU/L (IQR 1.6-10.1) in the women with a history of and active Graves, respectively. In the second trimester, TRAb titers were 0.9 IU/L (IQR 0.0-2.6) vs 2.3 IU/L (IQR 0.8-5.2), and in the third trimester, they declined to 0.6 IU/L (IQR 0.0-1.9) and 1.6 IU/L (IQR 0.5-4.0), respectively. From the first to the second trimester, TRAb titers declined by 18% in the history of Graves group, followed by an additional 33% reduction from the second to the third trimester. Similar patterns were observed in the active Graves group, with a 44% decline from the first to the second trimester, followed by an additional 28% reduction by the third trimester. The reduction in TRAb titers did not significantly differ between the active and the history of Graves groups.

**Table 3:** Thresholds of maternal serum thyrotropin receptor antibodies in trimesters 1, 2, and 3 for detecting fetal/neonatal hyperthyroidism

Trimester	TRAb, median (IQR)	AUC	Cutoff, IU/L	Sensitivity	Specificity	PPV%	NPV%	x [*] ULN
**History of Graves**								
1	1.05 (0.30-2.97)*^a,b^*	0.989 (0.979-1.000)	27	100	97	43	100	24.5
2	0.90 (0.01-2.60)*^c^*	0.998 (0.995-1.000)	21	100	99	83	100	19.1
3	0.62 (0.01-1.90)	0.996 (0.988-1.000)	7	100	96	39	100	6.3
**Active Graves**								
1	4.00 (1.60-10.12)*^d,e,f^*	0.907 (0.828-0.987)	11	89	84	29	99*	10.0
2	2.26 (0.83-5.16)*^g^*	0.955 (0.909-1.000)	13	86	96	60	99*	11.8
3	1.63 (0.52-4.04)*^h^*	0.959 (0.913-1.000)	11	83	97	71	99*	10.0

*x*[*] ULN represents the multiple of the assay-specific upper limit of normal corresponding to the TRAb cutoff value; *NPV would be 100% after excluding cases (n = 2) in which mothers received antithyroid drug treatment during pregnancy.

Abbreviations: AUC, area under the curve; IQR, interquartile range; NPV, negative predictive value; PPV, positive predictive value; TRAb, thyrotropin receptor antibody; ULN, upper limit of normal.

^*a*^Comparison of TRAb titers between trimester 1 and trimester 2 in history of Graves with *P* less than .01.

^*b*^Comparison of TRAb titers between trimester 1 and trimester 3 in history of Graves with *P* less than .001.

^*c*^Comparison of TRAb titers between trimester 2 and trimester 3 in history of Graves with *P* less than .01.

^*d*^Comparison of TRAb titers between trimester 1 and trimester 2 in active Graves with *P* less than .01.

^*e*^Comparison of TRAb titers between trimester 1 and trimester 3 in active Graves with *P* less than .001.

^*f*^Comparison of TRAb titers in trimester 1 between active and history of Graves with *P* less than .001.

^*g*^Comparison of TRAb titers in trimester 2 between active and history of Graves with *P* less than .001.

^*h*^Comparison of TRAb titers in trimester 3 between active and history of Graves with *P* less than .001.

### Thyrotropin receptor antibody titers according to diagnosis

**Figure 1:**
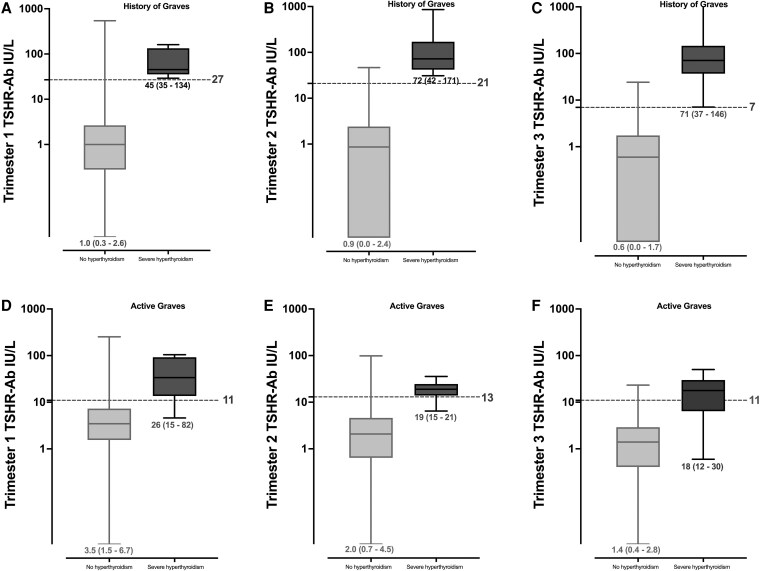
Maternal thyrotropin receptor antibody (TRAb) titers according to fetal/neonatal hyperthyroidism in relation to maternal diagnosis of a history of or active Graves. Box-and-whisker plots showing maternal TRAb titers according to neonatal outcome. A to C represent pregnancies in women with a history of Graves disease, and D to F represent pregnancies in women with active Graves disease. A and D correspond to the first trimester, B and E to the second trimester, and C and F to the third trimester. In all panels, pregnancies that resulted in fetal or neonatal hyperthyroidism or TRAb-mediated hypothyroidism (dark gray boxes) had significantly higher maternal TRAb titers compared with pregnancies without neonatal thyroid dysfunction (light gray boxes) (*P* < .001). Two patients in the active Graves disease group (D-F) had TRAb titers below the threshold value but their neonates developed hyperthyroidism; both mothers were treated with antithyroid drugs at the time of TRAb measurement.

TRAb titers were consistently elevated (*P* < 0.001) in pregnancies that subsequently developed fetal/neonatal hyperthyroidism (Fig. 1). Remarkably, an analysis of patients with a history of Graves who gave birth to neonates with fetal/neonatal hyperthyroidism showed that all 13 women had undergone treatment with radioactive iodine (I-131) prior to pregnancy (data not shown).

### History of Graves disease

#### Fetal/neonatal outcomes

Within the cohort with a history of Graves, 2.6% (13/500) of pregnancies encountered fetal/neonatal hyperthyroidism (Table 4). Twelve had hyperthyroidism, of whom 11 required treatment. Seven of these cases were diagnosed with fetal hyperthyroidism, resulting in one fetal death and one neonatal death during a cesarean delivery performed due to fetal distress. Additionally, there was one case of second-trimester pregnancy loss where TRAb titers were 545 IU/L in the first trimester and 21 IU/L in the second trimester. Ultrasonography evaluation at 16 + 4 w/d revealed fetal tachycardia, ascites, and pericardial effusion, indicative of fetal hyperthyroidism. Lastly, one neonate was diagnosed with hypothyroidism, probably attributed to blocking TRAbs, and was subsequently treated with T4. Although we used a total TRAb assay to determine the TRAb titers, this assay cannot differentiate between stimulating and blocking antibodies. However, the clinical presentation of hypothyroidism suggested the presence of blocking antibodies. The mother of this neonate was treated with T4 for her history of Graves throughout pregnancy and had a TRAb titer of 857 IU/L in the second trimester and 1050 IU/L in the third trimester. Seven neonates had subclinical hyperthyroidism, none of whom required further treatment.

**Table 4:** Fetal/neonatal thyroid function according to maternal diagnosis of a history of or active Graves

	History of Graves	Active Graves
**Number of patients**	**500**	**178**
**Euthyroid** (n, %)	450 (90.0)	130 (73.0)
**Fetal/neonatal hyperthyroidism**	13 (2.6)*^b^*	12 (6.7)
Hyperthyroidism requiring treatment (n, %)	11 (2.2)	4 (2.2)
Fetal hyperthyroidism	7	—
Neonatal hyperthyroidism	4	4
Hyperthyroidism, untreated (n, %)	1 (0.2)	6 (3.4)
Late hyperthyroidism, untreated (n, %)		1 (0.6)
Hypothyroidism due to blocking antibodies (n, %)	1 (0.2)	—
Hyperthyroidism followed by central hypothyroidism*^a^* (n, %)	—	1 (0.6)
**Subclinical hyperthyroidism** (n, %)	7 (1.4)	2 (1.1)
**Hypothyroidism** (n, %)	—	3 (1.7)
**Central hypothyroidism** (n, %)	—	2 (1.1)
**Transient hypothyroidism** (n, %)	—	5 (2.8)
**Subclinical hypothyroidism** (n, %)	19 (3.8)	5 (2.8)
**Transient subclinical hypothyroidism** (n, %)	1 (0.2)	10 (5.6)
**Solely increased fT4** (n, %)	4 (0.8)	4 (2.2)
**Transient solely increased fT4** (n, %)	6 (1.2)	5 (2.8)

Bold values indicate total number of participants (N) per group.

Abbreviations: fT4, free thyroxine; IQR, interquartile range; TSH, thyrotropin.

^*a*^This neonate, initially presenting with low TSH and elevated fT4 levels, subsequently exhibited low TSH and low fT4levels and required treatment with T4. The initial elevation in fT4 is most likely attributable to transplacental passage of maternal fT4, rather than neonatal hyperthyroidism.

^*b*^ *P* less than .05.

#### Thyrotropin receptor antibody threshold titers for fetal/neonatal hyperthyroidism

**Figure 2:**
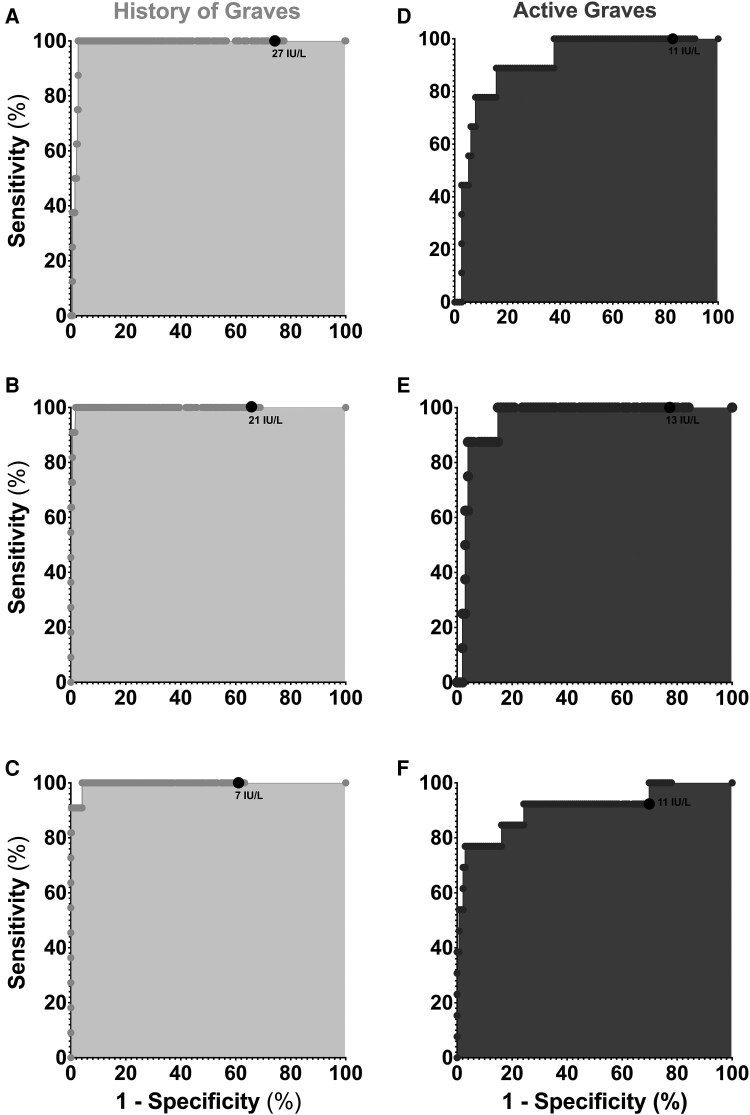
Predictive performance of trimester-specific thyrotropin receptor antibody (TRAb) threshold titers for fetal/neonatal hyperthyroidism in pregnancies with a history of or active Graves. Receiver operating characteristic curves (ROCs) showing sensitivity vs 1−specificity for TRAb threshold titers across pregnancy. A to C represent pregnancies in women with a history of Graves disease, and D to F represent pregnancies in women with active Graves disease. A and D correspond to the first trimester, B and E to the second trimester, and C and F to the third trimester. In the history of Graves disease group, TRAb threshold titers of A, 27 IU/L; B, 21 IU/L; and C, 7 IU/L corresponded to a maximal sensitivity of 100%. In the active Graves disease group, TRAb threshold titers of D, 11 IU/L; E, 13 IU/L; and F, 11 IU/L showed sensitivities of 89%, 86%, and 83%, respectively.

The history of Graves group demonstrated remarkable predictive ability for fetal/neonatal hyperthyroidism, as evidenced by high AUC values of 0.989, 0.998, and 0.996 across trimesters (Fig. 2 and Table 3). In the respective trimesters, a TRAb threshold titer of 27 IU/L, 21 IU/L, and 7 IU/L, respectively, corresponded with a maximal sensitivity of 100%, with a corresponding specificity ranging from 96% to 99%.

#### Clinical outcomes across trimesters according to thyrotropin receptor antibody threshold titers

A total of 8 of the 16 (50%) patients with TRAb titers of 27 IU/L or greater in the first trimester manifested fetal/neonatal hyperthyroidism; corresponding trimester-specific predictive values are shown in Table 3, with 4 fetuses already diagnosed in utero and 6 necessitating therapy. Conversely, among the 325 patients with TRAb titers less than 27 IU/L, no cases with fetal/neonatal hyperthyroidism were identified. Within this subset, 3 neonates were diagnosed with subclinical hyperthyroidism with none warranting therapy. During the second trimester, 11 out of 16 (69%) patients with TRAb titers of 21 IU/L or greater exhibited fetal/neonatal hyperthyroidism. Among these cases, 10 required treatment for hyperthyroidism, and 1 neonate developed hypothyroidism probably due to blocking antibodies. Among the 312 patients with TRAb titers less than 21 IU/L, no cases of fetal/neonatal hyperthyroidism were found.

In the third trimester, a total of 28 patients exhibited TRAb titers of 7 IU/L or greater, and among them, 11 (39%) experienced fetal/neonatal hyperthyroidism with 9 warranting therapy. Among the 387 patients with TRAb titers less than 7 IU/L, no hyperthyroidism-related complications occurred.

#### Change/rise in thyrotropin receptor antibody titers

None of the patients who started with TRAb titers less than 27 IU/L in the first trimester ended up with TRAb titers of 21 IU/L or greater in the second. However, 8 patients who had TRAb titers less than 27 IU/L in the first trimester displayed TRAb titers greater than 7 IU/L in the third trimester. Among these cases, 4 pregnancies proceeded uneventfully, 3 were diagnosed with subclinical hyperthyroidism with none warranting therapy, and 1 neonate developed a subclinical hypothyroidism, for which 3-month postpartum T4 was started. It is not likely that TRAbs played a role in this latter case.

### Active Graves disease

#### Fetal/neonatal outcomes

In the active Graves group, 12 of 178 pregnancies (7%) resulted in neonatal hyperthyroidism, 4 of whom were severe enough to require antithyroid treatment, with all 4 born to mothers who continued antithyroid therapy until delivery (see Table 4). Among these, 2 initially presented with subclinical hyperthyroidism at birth that progressed to overt hyperthyroidism, while the other 2 developed late-onset hyperthyroidism. The remaining 8 neonates were diagnosed with hyperthyroidism that did not require treatment; 7 of these 8 neonates were born to mothers who were on antithyroid drugs during the third trimester, and they exhibited late-onset hyperthyroidism after the maternal antithyroid drugs were metabolized and excreted post partum. The neonate whose mother abstained from antithyroid therapy during the third trimester exhibited hyperthyroidism directly at birth.

#### Thyrotropin receptor antibody threshold titers for fetal/neonatal hyperthyroidism

In predicting fetal/neonatal hyperthyroidism in the active Graves group (see Fig. 2 and Table 3), TRAbs showed statistically significant discriminatory power throughout pregnancy. The AUC values were impressive across all trimesters, with scores of 0.907 in the first trimester, 0.955 in the second, and 0.959 in the third. To determine appropriate TRAb threshold titers for classification, median threshold titers were derived from the active Graves group, setting a threshold titer at 11 IU/L for the first and third trimesters, and 13 IU/L for the second trimester. These threshold titers yielded high sensitivity and specificity: 89% and 84% for the first, 86% and 99% for the second, and 83% and of 99% for the third trimester, respectively. Adjusting these values for antithyroid drug use at the time of measurement increases the specificity to 100%, ensuring that all pregnancies at risk are adequately monitored.

#### Clinical outcomes across trimesters according to thyrotropin receptor antibody threshold titers

In the first trimester, 8 of the 28 (29%) patients with TRAb titers of 11 IU/L or greater experienced neonatal hyperthyroidism. Of these 8, 3 neonates required treatment for hyperthyroidism and 5 did not necessitate treatment. In stark contrast, within the cohort where TRAb titers were less than 11 IU/L, comprising 95 patients, only 1 case (1%) of late transient neonatal hyperthyroidism was reported. This condition manifested on the eighth day post partum but no medical interventions were necessary. Notably, the mother of this neonate underwent continuous antithyroid drugs therapy throughout her pregnancy.

In the second trimester, 10 patients had TRAb titers of 13 IU/L or greater and 6 (60%) of them developed fetal/neonatal hyperthyroidism. Of these 6, 3 neonates required treatment for hyperthyroidism, for 2 treatment was not necessary, and 1 initially presented with biochemical hyperthyroidism early post partum that evolved into central hypothyroidism during further follow-up. The mother of the latter neonate showed a severe hyperthyroid state throughout pregnancy. Again, 1 out of 98 patients (1%) developed hyperthyroidism despite a TRAb titer of less than 13 IU/L. No treatment was necessary for this latter neonate, and his mother underwent continuous antithyroid drugs therapy throughout her pregnancy. She showed a drop of her TRAb titer from 11.9 IU/L in trimester 1 to 6.5 IU/L in the second.

In the third trimester, 10 of the 14 (71%) patients who exhibited TRAb titers of 11 IU/L or greater experienced fetal/neonatal hyperthyroidism. Four of these patient cases necessitated treatment for observed hyperthyroidism, and one developed central hypothyroidism due to inadequate control of maternal hyperthyroidism in the third trimester. The remaining 5 patients were diagnosed with hyperthyroidism but did not require antithyroid drugs according to the treating pediatrician. Despite TRAb titers below the threshold of 11 IU/L in the third trimester, 2 of 134 (1%) neonates exhibited hyperthyroidism. Neither of these two neonates required treatment. Importantly, both mothers were treated with antithyroid drugs throughout the third trimester.

#### Rise in thyrotropin receptor antibody titer

Within the active Graves cohort, no patient had TRAb titers less than 11 IU/L in the first trimester and above the TRAb titer threshold of 13 IU/L in the second trimester. When comparing the first and third trimester, however, we identified one patient whose TRAb titer was less than 11 IU/L in the first trimester (4.6 IU/L) and surpassed the threshold in the third trimester (16.9 IU/L); no measurement was available in the second trimester. This patient gave birth to a neonate who developed delayed hyperthyroidism on the eighth day post partum, but no therapy was required.

#### Conversion factors and test standardization for different assays

**Figure 3:**
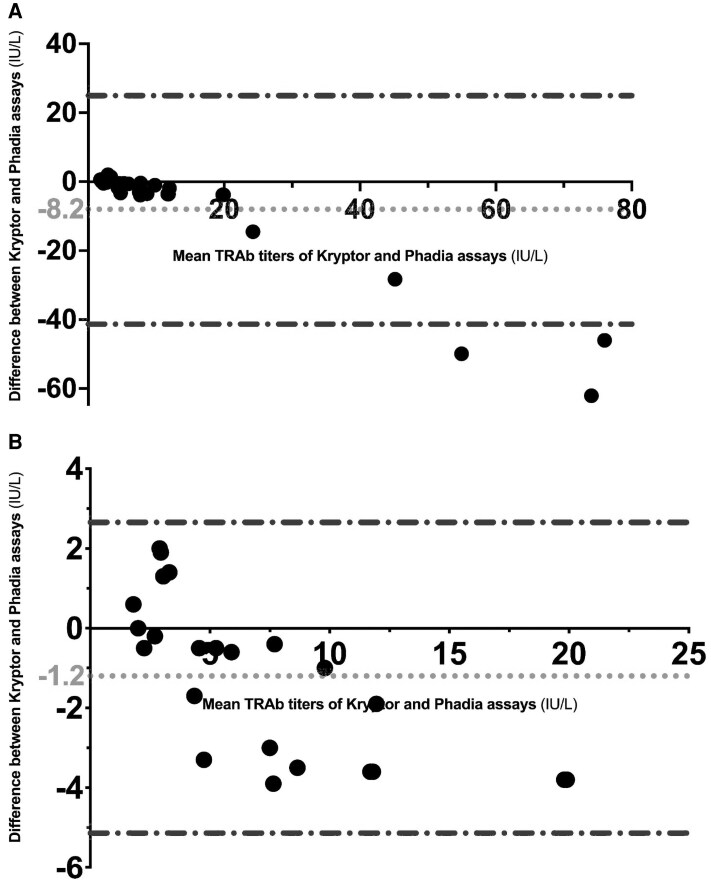
Bland-Altman plot comparing thyrotropin receptor antibody (TRAb) titers measured by Kryptor vs Phadia TRAb assays. A and B show Bland-Altman plots of the difference between TRAb titers measured with the Kryptor and Phadia assays (Kryptor minus Phadia, y-axis) plotted against the mean concentration of the two measurements (x-axis), expressed in IU/L. The dashed light gray line represents the mean bias and the dashed-dotted dark gray lines indicate the 95% limits of agreement. A shows the full measurement range, with a mean bias of −8.2 IU/L (SD 17) and limits of agreement from −41 to +25 IU/L. B shows the clinically relevant range (<27 IU/L), in which agreement between assays was markedly improved (mean bias −1.2 IU/L; SD 2.0; 95% limits of agreement −5.1 to +2.7 IU/L).

The Phadia assay produced systematically lower TRAb titers than the Kryptor, with an overall mean bias of −8.2 IU/L (SD 17; 95% limits of agreement: −41 to +25 IU/L) (Fig. 3). When the analysis was restricted to TRAb titers below 27 IU/L, a range below which no fetal or neonatal hyperthyroidism was observed, the bias diminished to −1.2 IU/L (SD 2.0; 95% limits of agreement: −5.1 to +2.7 IU/L), indicating close agreement within this range.

## Discussion

Our study, encompassing to our knowledge the largest cohort to date, demonstrates that trimester-specific TRAb titers provide valuable predictive insights into fetal/neonatal hyperthyroidism in pregnancies complicated by Graves disease. For women with a history of Graves, we identified TRAb threshold titers of 27 IU/L, 21 IU/L, and 7 IU/L for the first, second, and third trimesters, respectively. All patient cases of fetal or neonatal hyperthyroidism in women with a history of Graves disease occurred after prior radioactive iodine therapy, consistent with earlier reports of early-onset fetal Graves disease in this subgroup (10). Notably, these values achieved a negative predictive value of 100%, allowing safe exclusion of fetal/neonatal complications. In addition, this suggests that repetitive TRAb measurements are not necessary when first-trimester TRAb titers are below the established thresholds, reducing the need for repeated testing and unnecessary monitoring. The observed decline in TRAb titers across gestation is consistent with previous observations that antibody concentrations tend to decrease during the second half of pregnancy, most likely due to the generalized immunosuppressive state that accompanies gestation (11). This physiological modulation of maternal immunity likely contributes to the attenuation of autoimmune activity and may explain the progressive fall in TRAb levels observed in our cohort.

In contrast, among women with active Graves, lower trimester-specific TRAb threshold titers of 11 IU/L, 13 IU/L, and 11 IU/L were identified. Although these TRAb thresholds excluded disease in most cases, neonatal hyperthyroidism still occurred in some women who were treated by antithyroid drugs. This suggests that antithyroid treatment may reduce antibody activity or impair placental transfer, limiting sensitivity. Continued fetal surveillance by determination of fetal heart rate and ultrasound is therefore recommended, regardless of actual maternal TRAb titers.

Our findings question the widespread practice, endorsed by both national and international guidelines, of applying a uniform threshold titer of 3.7 times the assay-specific ULN across all TRAb assays. This recommendation, based on a single systematic review, assumes uniform characteristics of all commercially available TRAb assays. Applied to our Kryptor assay (ULN: 1.1 IU/L), this would mean a TRAb threshold titer of 4.1 IU/L. In the history of Graves group, only 3% (16/500) exceeded a TRAb threshold of 27 IU/L, while use of a TRAb threshold titer of 4.1 IU/L recommended according to guidelines would increase this proportion to 15% (75/500). This latter case would result in a 5-fold rise in unnecessary tests and ultrasounds. In contrast, 34% (60/178) of patients with active Graves would be classified as high risk using the TRAb threshold titer of 4.1 IU/L, compared with 16% (28/178) using our TRAb threshold titer of 11 IU/L.

The Kryptor-derived TRAb threshold titer cannot be directly compared with results from other TRAb assays, given the current lack of harmonization among commercially available TRAb assays. This observation is in line with previous comparative studies demonstrating high diagnostic performance across TRAb assays but limited interchangeability due to substantial interassay variability (12). The quantitative discrepancy between the Kryptor and Phadia assays, as clearly illustrated in Fig. 3, shows the difficulties in directly comparing TRAb titers measured by different TRAb assays and emphasizes the necessity of establishing conversion factors for each TRAb assay to ensure consistent clinical interpretations. While often the ULN of a TRAb assay for comparison across systems is suggested in the literature (7), this practice carries inherent risks without adjustment for assay differences. For instance, our study used a Kryptor-derived TRAb threshold titer of 11 IU/L, corresponding to 10 times the ULN (11/1.1 IU/L). Applying the same factor to the Phadia ULN of 2.9 IU/L would suggest a TRAb threshold of 29 IU/L, significantly higher than Kryptor's 11 IU/L, despite the fact that the Phadia TRAb assay yields lower raw results. This discrepancy underscores the potential for inconsistent clinical interpretations and possibly unsafe outcomes when using ULN multiples across different TRAb assays without adjustment. Importantly, the widely applied practice of using fixed ULN-based multipliers originates largely from studies using first- and second-generation TRAb assays, and extrapolation of these thresholds to contemporary automated assays may result in serious misclassifications of fetal risk. We advocate recalibrating absolute values to a common international standard, thereby establishing conversion factors to enhance the reliability and safety of patient management across various testing platforms. Although recalibration to a common international standard is an important step toward harmonization, it will not necessarily eliminate interassay differences. Each assay platform has its own calibration characteristics, and the between-assay relationship may vary across the concentration range, implying that a single fixed conversion factor may be insufficient. This underscores the need for assay-specific validation for the intended clinical use. This comparative analysis was exploratory in nature and based on a limited sample size, but serves to illustrate that ULN-based TRAb thresholds associated with fetal or neonatal hyperthyroidism may substantially differ across assay platforms.

### Clinical implications

Our study refines the management of pregnancies complicated by Graves disease by establishing trimester-specific TRAb threshold titers that enable and improve risk stratification and substantially reduce unnecessary monitoring. These TRAb threshold titers should help to achieve a perfect negative predictive value in patients with a history of Graves, thereby eliminating the need for repeated testing and minimizing health-care costs and patient burden.

In patients with active Graves treated with antithyroid drugs, the sensitivity of TRAb testing is not absolute, as neonatal hyperthyroidism may still occur despite maternal TRAb titers below the trimester-specific TRAb thresholds. Therefore, irrespective of TRAb titers continued monitoring throughout pregnancy and post partum remains warranted in this subgroup. Finally, our findings challenge the actual guideline recommendation to apply a uniform TRAb threshold titer of 3.7 times the ULN across all TRAb assays. These recommendations regarding the timing of TRAb assessment are applicable only to the Kryptor TRAb assay; extrapolation to other TRAb platforms, even when calibrated against a common international standard, requires assay-specific clinical validation before implementation.

## Data Availability

Some or all datasets generated during and/or analyzed during the current study are not publicly available due to legal and informed consent restrictions but are available from the corresponding author on reasonable request.

## Abbreviations

AUC: area under the curve
CI: confidence interval
fT4: free thyroxine
IQR: interquartile range
NPV: negative predictive value
OR: odds ratio
PPV: positive predictive value
ROC: receiver operating characteristic curve
SD: standard deviation
TRAb: thyroid-stimulating hormone receptor antibodies
TSH: thyroid-stimulating hormone
ULN: upper limit of normal
